# Quercetin Antagonizes the Sedative Effects of Linalool, Possibly through the GABAergic Interaction Pathway

**DOI:** 10.3390/molecules28145616

**Published:** 2023-07-24

**Authors:** Mehedi Hasan Bappi, Abdullah Al Shamsh Prottay, Hossam Kamli, Fatema Akter Sonia, Md. Nayem Mia, Md. Showkoth Akbor, Md. Munnaf Hossen, Samir Awadallah, Mohammad S. Mubarak, Muhammad Torequl Islam

**Affiliations:** 1 Department of Pharmacy, Bangabandhu Sheikh Mujibur Rahman Science and Technology University, Gopalganj 8100, Bangladesh; mehedibappi22@gmail.com (M.H.B.); abdullah103637@gmail.com (A.A.S.P.); bsmrstufatema@gmail.com (F.A.S.); nayemnijom@gmail.com (M.N.M.); showkothakbor3@gmail.com (M.S.A.); 2 Department of Clinical Laboratory Sciences, College of Applied Medical Sciences, King Khalid University, Abha 61421, Saudi Arabia; hmkamli@kku.edu.sa; 3 School of Health and Biomedical Sciences, RMIT University, Bundoora, VIC 3083, Australia; mmhossen_nfs@yahoo.com; 4 Department of Medical Lab Sciences, Faculty of Allied Medical Sciences, Zarqa University, Zarqa 13110, Jordan; sawadallah@zu.edu.jo; 5 Department of Chemistry, The University of Jordan, Amman 11942, Jordan

**Keywords:** sedative modulatory activity, quercetin, linalool, GABA_A_ receptor, molecular docking simulation

## Abstract

Sedatives promote calmness or sleepiness during surgery or severely stressful events. In addition, depression is a mental health issue that negatively affects emotional well-being. A group of drugs called anti-depressants is used to treat major depressive illnesses. The aim of the present work was to evaluate the effects of quercetin (QUR) and linalool (LIN) on thiopental sodium (TS)-induced sleeping mice and to investigate the combined effects of these compounds using a conventional co-treatment strategy and *in silico* studies. For this, the TS-induced sleeping mice were monitored to compare the occurrence, latency, and duration of the sleep-in response to QUR (10, 25, 50 mg/kg), LIN (10, 25, 50 mg/kg), and diazepam (DZP, 3 mg/kg, i.p.). Moreover, an in silico investigation was undertaken to assess this study’s putative modulatory sedation mechanism. For this, we observed the ability of test and standard medications to interact with various gamma-aminobutyric acid A receptor (GABA_A_) subunits. Results revealed that QUR and LIN cause dose-dependent antidepressant-like and sedative-like effects in animals, respectively. In addition, QUR-50 mg/kg and LIN-50 mg/kg and/or DZP-3 mg/kg combined were associated with an increased latency period and reduced sleeping times in animals. Results of the in silico studies demonstrated that QUR has better binding interaction with GABA_A_ α3, β1, and γ2 subunits when compared with DZP, whereas LIN showed moderate affinity with the GABA_A_ receptor. Taken together, the sleep duration of LIN and DZP is opposed by QUR in TS-induced sleeping mice, suggesting that QUR may be responsible for providing sedation-antagonizing effects through the GABAergic interaction pathway.

## 1. Introduction

Sleep disorders are conditions that prevent us from getting adequate sleep. Approximately 1 billion individuals worldwide have sleep issues, and 50% are over 65 [1]. These problems are associated with physical and psychological issues that may lead to depression [2]. On the other hand, depression is a prevalent and complicated mental condition that affects emotional well-being [3]. The onset of depression may be influenced by various variables, including obesity, neurological and mental problems, and inflammatory diseases [4]. To treat sleep disorders, some drugs are required in addition to changes in nocturnal sleep habits, the practice of sleep hygiene, and physical activity [5]. For instance, modafinil is an excellent medicine for the management of hypersomnia [6]. Treatment of mild obstructive sleep apnea (OSA) and hypopnea involves maintaining nasal patency [7]. However, a third of depressed people experience treatment-resistant depression after taking medication [8]. Most sedative–hypnotic medications have a wide range of undesirable side effects, including memory loss, cognitive dysfunction, and discontinuation syndrome [9]. Although antidepressant medications lessen abnormal brain activity, they may have undesired adverse effects [4].

The World Health Organization (WHO) listed major depressive disorder (MDD), a recurrent neuropsychiatric disease, as the third biggest contributor to the global disease burden in 2008 and predicted that it will take the top spot by 2030; it is one of the most common causes of social and economic stress [10]. Moreover, MDD increases the possibility of developing other chronic diseases that might cause further impairment or fatality in addition to causing an elevated risk of individual distress and suicidal thoughts [11]. Research findings indicate that the pathophysiology of depression is significantly influenced by GABAergic transmission disruption [12]. Alterations in certain GABAergic subtypes and dysregulation of GABA neurotransmission in MDD patients and animal models of depression are mainly caused by stress or genetics [13]. In this respect, a variety of regional neurotransmitter frameworks, including the glutamatergic excitatory counterpart, are modulated by GABA [14]. The most widely recognized receptor, GABA_A_, has been thoroughly defined as the target of several psychotropic substances, including benzodiazepines, ethanol, and barbiturates [15]. On the other hand, the major GABA_A_ receptor isoform is made up of two α-subtypes, two β-subtypes, and one γ-subtype, or one δ-subtype. In the adult brain, 90% of GABA_A_ receptors are γ-containing receptors, which are mostly distributed in synaptic locations [16]. However, it is generally accepted that the primary mature isoform consists of α1, β2, and γ2 subunits, which are positioned as γ2β2α1β2α1 counterclockwise around a central pore when observed from the outside of the cell [17].

Additionally, the benzodiazepine category of sedative medicines targets the ionotropic GABA_A_ receptor protein complex [18]. GABA_A_ receptors must have both α and γ subunits, which the benzodiazepine binds to, for them to be susceptible to the effects of these drugs [19]. Although almost all GABA_A_ receptors (those carrying subunits α1, α2, α3, or α5) are responsive to benzodiazepines, there are certain GABA_A_ receptors (subunits α4 or α6) that are immune to traditional 1,4-benzodiazepines [20]. In contrast to those with greater activity at GABA_A_ receptors, including the α2 and/or α3 subunits, benzodiazepine receptor ligands with stronger potential at the α1 and/or α5 appear to have greater anxiolytic effects [21]. Furthermore, there is a correlation between GABRB2 and GABRG2 that results in the downregulation of GABA_A_ receptor activity in people with schizophrenia, bipolar illness, and idiopathic generalized epilepsies. When combined, GABRA1 (increasing) and GABRB2/GABRG2 (decreasing) variations appear to have opposite impacts on the GABA_A_ receptor activation in people [22].

Multiple neurological disorders are characterized by imbalanced GABAergic transmission [23]. Epilepsy may take many different forms when there is an asymmetry between excitement and inhibition brought on by faulty GABAergic transmission [24]. Both epileptic humans and animal models have shown certain mutations in the genes encoding the α1, α6, β2, β3, γ2, or δ subunits of the GABA_A_ receptor [25]. According to published reports, GABA_A_ receptors appear to be a promising therapeutic target for the treatment of Alzheimer’s disease (AD) [26,27]. A recent study found that adult-onset cervical dystonia (CD) patients have lower GABA levels in the right thalamus and that the presence of GABA_A_ receptors is inversely connected to the extent of the disease and the degree of the dystonia [28]. In another study, the pathophysiology of autism spectrum disorder (ASD) was characterized by an imbalance in glutamatergic/GABAergic signaling pathways and neuroinflammatory processes, which were also seen in a number of ASD mouse models [29]. One of the defining characteristics underlying behavioral abnormalities in autism is the imbalance between excitatory and inhibitory transmission brought about by differences in GABA levels [30]. Cortical GABA_A_ receptor inhibition impairs decision-making, social behavior, and attention, according to research [31]. Therefore, it is vital to produce medications to treat these neurological illnesses.

**Figure 1:**
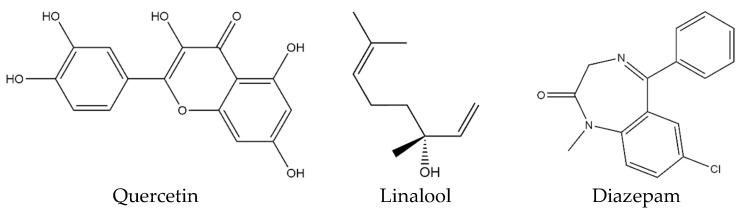
Chemical structure of quercetin, linalool, and diazepam.

Natural products, especially plant-derived constituents, have promising neuroprotective properties that can effectively help to prevent and treat depression without any side effects [32]. Quercetin (QUR) (Figure 1) is the most prevalent polyphenolic flavonoid, is found in fruits and vegetables such as apples, berries, citrus fruits, grapes, cherries, leafy greens, green tea, capers, etc., and exhibits a broad spectrum of health-promoting actions in diseases [33,34,35]. According to research, QUR may promote neurogenesis and the restoration of nerve tissue [36]. Since QUR functions by defending the tissue against oxidative stress caused by, or arising from, physiological metabolism, its neuroprotective action is essentially connected to its anti-inflammatory and antioxidant capabilities [37]. Moreover, the combination of QUR and mesenchymal stromal cell transplantation greatly reduced oxidative stress and cell apoptosis while also having a synergistic neuroprotective impact on spinal cord injuries [38]. On the other hand, linalool (LIN) and linalyl acetate, the primary components of lavender essential oil, and other essential oils like coriander oil, exhibit numerous important bioactivities, including sedative and hypnotic effects in experimental animals [39,40]. Furthermore, multiple in vitro and in vivo investigations have shown that LIN exhibits a wide variety of biological characteristics such as anti-inflammatory, anxiolytic, anticancer, antibacterial, antidepressant, hepatoprotective, and neuroprotective activities [41]. Research findings indicated that the sedative effects of *Cissus sicyoides* L. (Vitaceae) might be attributed to the presence of α-tocopherol, which works in conjunction with LIN and flavonoids to enhance the effects of sedatives [42]. Another behavioral study revealed that LIN has anxiolytic effects without impairing the animal’s ability to move around [43]. The antioxidant and anti-inflammatory properties of LIN are responsible for its neuroprotective actions on oxygen-glucose deprivation-induced neuronal injury [44].

Several studies were performed on the antidepressant effects of QUR [45] and LIN [46]. The sedative effects of LIN were also demonstrated in experimental animals [47]. There are bioactive compounds that have antidepressant and sedative effects, such as the essential oil (EO) of leaves from *Citrus limon* L. (Family: Rutaceae) [48] and thiophene derivatives [49]. Drugs with sedative but anti-depressive effects might be a good choice for sleep disturbances and associated coincidences [50]. Due to their high doses, sedative drugs may act as hypnotics and can impart some unavoidable side effects [51]. In contrast, at high doses, anti-depressant drugs also cause some serious adverse events such as GI disturbances, pain, anxiety, nausea, agitation, insomnia, and many more [52].

Combined drug therapy has gained much attention in the present era in many areas, including in oncology and neurobiological studies. This is because certain drugs have poor solubility, poor bioavailability, and high metabolic effects, resulting in low bioactivity. Combined strategies can improve solubility, bioavailability, and treatment response and minimize adverse events [53]. In general, QUR exists in its glucoside form in nature. Glucosidic QUR is unable to cross biological membranes. This is due to its low lipid solubility [54]. The terpene alcohol LIN has high lipophilicity and can readily cross the cell membrane [55]. Certain natural terpenes, including LIN, have penetration-enhancing capabilities [56]. Thus, LIN may enhance the biological membrane-crossing capability of QUR.

Based on the preceding discussion, this study aims to investigate the individual and combined effects of QUR and LIN (Figure 1) on TS-induced sleeping mice. Additionally, in silico studies were performed to understand the possible mechanism(s) of action for this neurological effect.

## 2. Results

### 2.1. In Vivo Study

The results in Table 1 suggest that both QUR (Gr-III to Gr-V) and LIN (Gr-VI to Gr-VIII) produce dose-dependent effects on the TS-induced animals. At all doses, LIN showed a lower latency period but a higher sleep duration than the QUR groups. QUR dose-dependently increased latency and decreased sleeping time in animals. The standard DZP (Gr-II) drug produced a better sedative effect than the QUR and LIN groups.

Listed in Table 2 are results related to the latent period and sleeping time in test groups and controls. The results reveal that the incidence of sleep among the animals was 100% in all groups. DZP-3 (Gr-IV) reduced latency time significantly (*p* < 0.05) when compared to the control group (Gr-I). Similarly, LIN-50 (Gr-III) reduced the latency compared to other groups (except Gr-I and IV); however, its effect was insignificant compared to Gr-I. On the other hand, QUR-50 (Gr-II) or its combinations with LIN-50 and DZP-3 (Gr-V and Gr-VI) increased the latency time in animals more than in the other groups, including Gr-I. In addition, results showed that QUR-50 (Gr-II) significantly modulated LIN-50 (Gr-V)’s latent period. Moreover, animals in Gr-III, IV, and VI had higher sleep duration compared to those in Gr-I. Finally, QUR-50 (Gr-II) significantly modulated LIN-50 (Gr-V)’s sleep duration.

The percentage modulations of latency and sleep duration in the tests and/or standard groups are shown in Table 3, compared to the vehicle (Gr-CI: control) group as shown in Table 2. Results show that DZP in Gr-CIV (DZP-3) only reduced animal reflex time by 92.23%. Other groups did not show a decrease in latency time compared to the control group (Gr-CI). In Gr-CIV, there was an increase in sleep duration (58.63%) compared to the control group (Gr-CI), which was then followed by Gr-CIII (LIN-50) and Gr-CVI (QUR-50 + DZP-3 + LIN-50). On the other hand, QUR-50 (Gr-CII) alone or combined with LIN-50 (Gr-CV) did not increase sleeping time in animals. However, a modulatory effect was observed in their combination with DZP-3 (Gr-CVI).

### 2.2. In Silico Study

#### 2.2.1. GABA Homology Model

**Figure 2:**
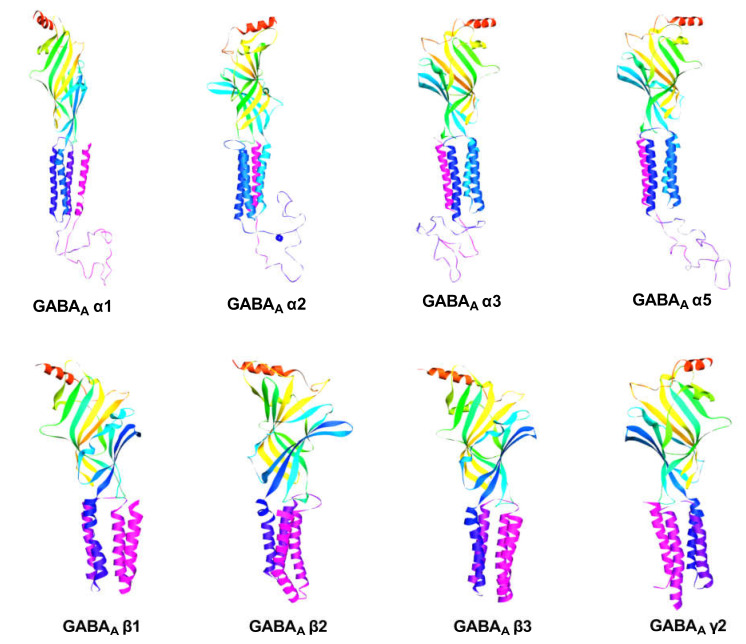
The homology model of human GABA_A_ (α1, α2, α3, α5, β1, β2, β3, and γ2) receptors through the SWISS-MODEL.

Homology modeling is one of the most effective techniques to computationally determine the 3D structure of a protein from its amino acid sequence [57]. It is carried out using a variety of programs and servers and includes several simple and easy procedures. This helps to identify innovative drug candidates, which is crucial for advancing, simplifying, and improving drug development [58]. In this study, the FASTA-formatted sequences of GABA_A_ receptor subunits were obtained from UniProt and used for further research. We have employed the SWISS-MODEL online server (https://swissmodel.expasy.org/interactive, accessed on 18 November 2022) to generate the best homology template for GABA_A_ (α1, α2, α3, α5, β1, β2, β3, and γ2) receptor subunits from the UniProt database (UniProt ID: P14867, P47869, P34903, P31644, P18505, P47870, P28472, and P18507, respectively), and their similar PDB (PDB ID: 6huj, 6hug, 6huj, 7qne, 6dw0, 6x3x, 7qn6, and 7qna, respectively), sequence of amino acids, which was reported to NCBI Blast Programs. Figure 2 shows a 3D homology model of GABA_A_ receptors. The GABA homology models were then refined by PyMOL version 1.7.4.5 Edu and optimized using the SWISS-PDB Viewer software tool (version 4.1.0). Validation of the predicted models of the GABA_A_ receptor subunits was performed by submitting PDB files to the PDBsum site by the PROCHECK server. The stereochemical properties of projected models were confirmed by the Phi/Psi Ramachandran plot.

**Figure 3:**
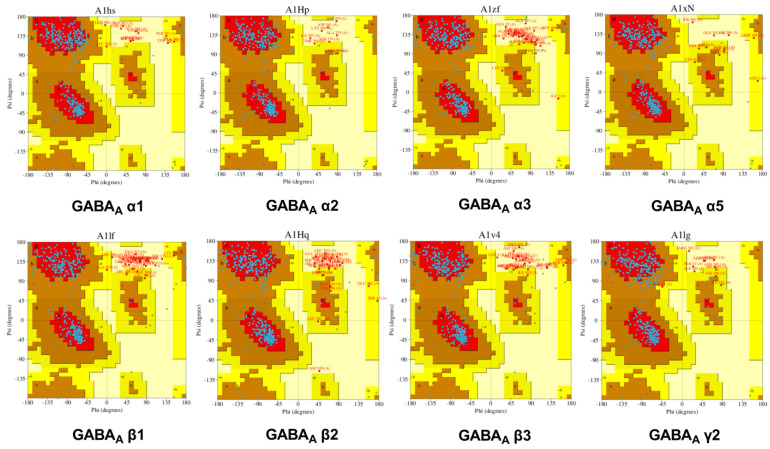
The optimized model of human GABA_A_ (α1, α2, α3, α5, β1, β2, β3, and γ2) receptors used by PROCHECK.

The Ramachandran plot is a straightforward method for observing the distribution of torsion angles in protein complexes. It also provides an overview of the torsion-angle values that are allowed and forbidden, which is crucial for determining the validity of the three-dimensional structures of proteins. The phi–psi torsion orientations of each residue in the structure are shown on the Ramachandran map (except those at the chain termini). As glycine residues are not limited to the plot areas designated by one of the additional side chain variants, they are depicted as triangles. The shading and coloration of the plot show numerous places: the “core” regions, which represent the most advantageous phipsi value combinations. The most preferred residues are shown in red; the permitted residues are shown in yellow, and the extensively allowed residues are shown in faint yellow. The white color indicates residues in the disallowed region. In an ideal scenario, those “core” sections would have included more than 90% of something like the remainder. One of the most reliable indicators of stereochemical integrity is the proportion of residues in “core” sites (Figure 3).

According to the Ramachandran plot statistics, residues in the most favored areas were around 93.86%, 93.80%, 91.35%, 92.61%, 96.91%, 96.72%, 91.12%, and 96.61% for GABA_A_ (α1, α2, α3, α5, β1, β2, β3, and γ2), respectively.

##### Interaction of QUR with GABA_A_ Receptor Subunits

**Figure 4:**
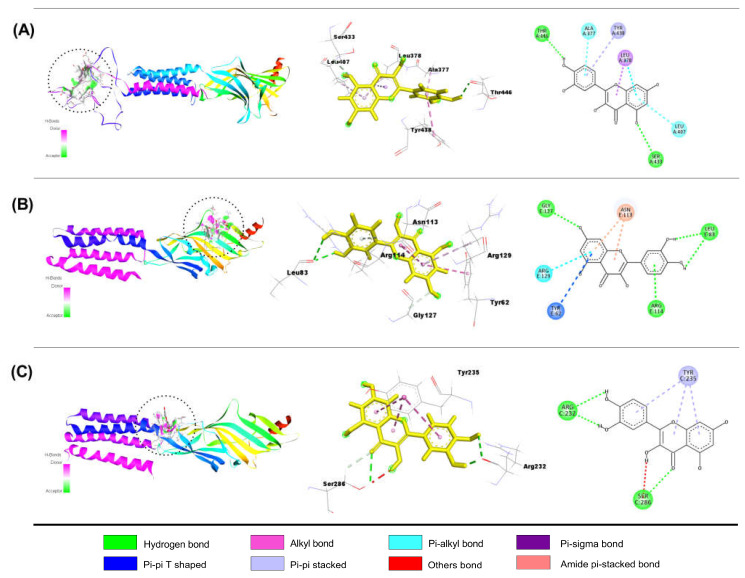
The best molecular docking interactions of GABA_A_ α3 (**A**); GABA_A_ β1 (**B**); and GABA_A_ γ2 (**C**) receptors with quercetin.

Table 4 shows the results of our molecular docking study (binding affinity (Kcal/mol, number of hydrogen, hydrophobic, and other bonds)) of QUR with GABA_A_ (α1, α2, α3, α5, β1, β2, β3, and γ2) receptor subunits. QUR demonstrated better binding affinities with GABA_A_ α3, GABA_A_ β1, and GABA_A_ γ2 receptor subunits than others. The binding values were − 8.2, − 8.0, and − 7.0 kcal/mol, respectively. Moreover, QUR connects to the GABA_A_ α3 subunit through one carbon-hydrogen (SER433) and one conventional hydrogen (TER446) bond, three pi-alkyls (ALA377, LEU378, LEU407), one pi-sigma (LEU378), and one pi-pi stacking bond (TYR438). QUR also binds to the GABA_A_ β1 subunit through two conventional hydrogens (LEU83), one carbon-hydrogen (GLY127), one pi-donor hydrogen (ARG114) bond, one pi-alkyl (ARG129), one pi-pi T shaped (TYR62), and two amide pi-stacked (ASN113) bonds. Furthermore, QUR was linked to the GABA γ2 subunit by three conventional H-bonds (SER286, ARG232, ARG232), one carbon-hydrogen (SER286) bond, and three pi-pi stacked bonds (TYR235). Figure 4A–C depicts the 2D and 3D structures of QUR non-bond interactions with GABA_A_ α3 (A), GABA_A_ β1 (B), and GABA_A_ **γ**2 (C) receptor subunits.

##### Interaction of LIN with GABA_A_ Receptor Subunits

**Figure 5:**
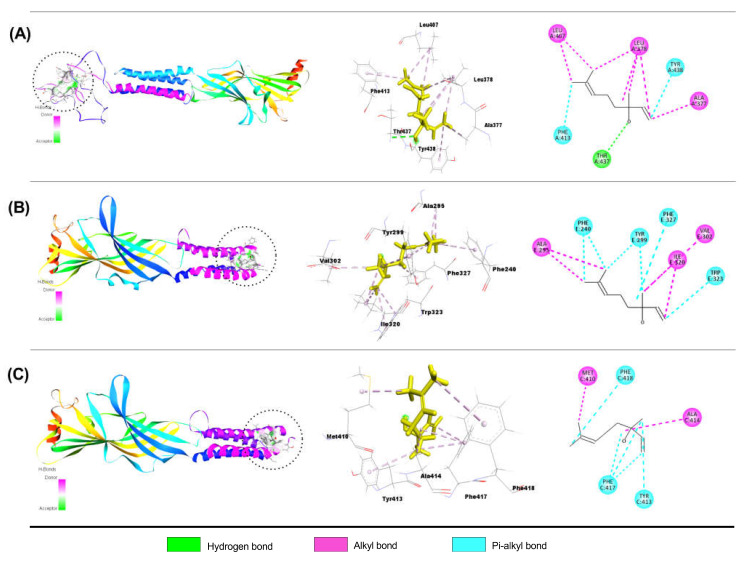
The best molecular docking interaction of GABA_A_ α3 (**A**); GABA_A_ β1 (**B**); and GABA_A_ γ2 (**C**) receptors with linalool.

Table 5 lists the results of our molecular docking study (binding affinity (Kcal/mol, number of hydrogen, hydrophobic, and other bonds)) of LIN with GABA_A_ (α1, α2, α3, α5, β1, β2, β3, and γ2) receptors subunits. The results revealed that LIN shows better binding affinities with GABA_A_ α3, GABA_A_ β1, and GABA_A_ γ2 receptor subunits than others. The binding values were −5.2, −5.8, and −4.3 kcal/mol, respectively. In comparison with QUR, LIN exhibited moderate docking values with GABA_A_ receptor subunits (α3, β1, and γ2). Results also showed that LIN interacts with GABA_A_ α3 subunit via one conventional hydrogen bond (THR437), seven alkyls (ALA377, LEU378, LEU378, LEU407, LEU378, LEU407, and LEU378), and two pi-alkyl bonds (PHE413, TYR438). LIN also attaches to the GABA_A_ β1 subunit via five alkyls (ALA295, ALA295, ILE320, VAL302, and ILE320), and seven pi-alkyl bonds (PHE240, PHE240, TYR299, TYR299, TRP323, TRP323, and PHE327). Furthermore, LIN has linked to the GABA_A_ γ2 subunit via two alkyls (ALA414, MET410) and five pi-alkyls (TYR413, PHE414, PHE414, PHE414, and PHE418) bonds. Figure 5A–C depicts the 2D and 3D structures of LIN non-bond interactions with GABA_A_ α3 (A), GABA_A_ β1 (B), and GABA_A_ γ2 (C) receptor subunits.

##### Interaction of DZP with GABA_A_ Receptor Subunits

**Figure 6:**
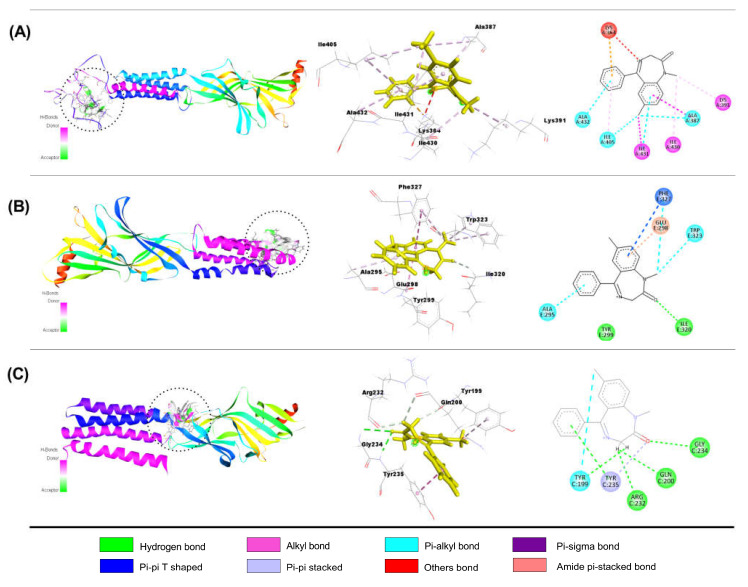
The best molecular docking interaction of GABA_A_ α3 (**A**); GABA_A_ β1 (**B**); and GABA_A_ γ2 (**C**) receptors with diazepam.

Listed in Table 6 are the results of the molecular docking study (binding affinity (Kcal/mol, number of hydrogen, hydrophobic, and other bonds)) of DZP with GABA_A_ (α1, α2, α3, α5, β1, β2, β3, and γ2) receptors subunits. DZP demonstrated better binding affinities with GABA_A_ α3, GABA_A_ β1, and GABA_A_ γ2 receptor subunits than others; the binding values were −6.8, −7.8, and −7.7 kcal/mol, respectively. Compared to DZP, QUR exhibited higher docking values with GABA_A_ receptor subunits (α3 and β1). Results showed that DZP connects to the GABA_A_ α3 subunit through one pi-cation (LYS364), five alkyls (ALA387, ILE405, ILE431, LYS391, ILE430), and four pi-alkyls (ILE405, ALA432, ALA387, ILE431). DZP also binds to the GABA_A_ β1 subunit through one carbon-hydrogen (ILE320), one pi-pi T-shaped (PHE327), one amide pi-stacked (GLU298) bond, and four pi-alkyls (TRP323, TRP323, PHE327, ALA295). Furthermore, DZP is linked to the GABA_A_ γ2 subunit by two conventional hydrogens (GLY234, TYR235) and three carbon-hydrogen (TYR199, GLN200, ARG232) bonds, one pi-pi stacked (TYR235), and one pi-alkyl (TYR199) bond. Shown in Figure 6 (A, B, and C) are the 2D and 3D structures of DZP non-bond interactions with GABA_A_ α3 (A), GABA_A_ β1 (B), and GABA_A_ γ2 (C) receptor subunits.

#### 2.2.2. Pharmacokinetics and Drug-Likeness Properties

**Figure 7:**
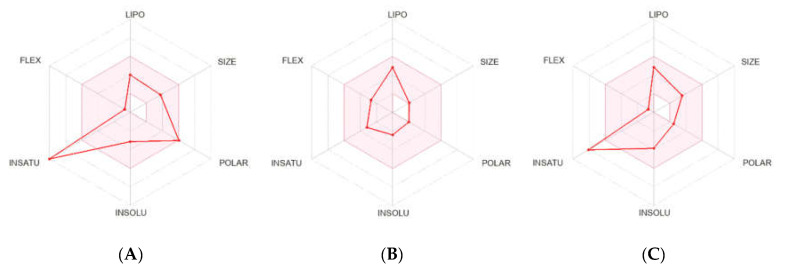
ADMET properties of (**A**) quercetin, (**B**) linalool; and (**C**) diazepam generated by SWISS-ADME [The colored zone is the suitable physicochemical space for oral bioavailability. LIPO (Lipophilicity): −0.7 < XLOGP3 < + 5.0; SIZE: 150 g/mol < MV < 500 g/mol; POLAR (Polarity): 20 Å^2^ < TPSA < 130 Å^2^; INSOLU (Insolubility): − 6 < Log S (ESOL) < 0; INSATU (Insaturation): 0.25 < Fraction Csp 3 < 1; FLEX (Flexibility): 0 < NUM; rotatable bonds < 9].

We used the SWISS-ADME online application [59] in our *in silico* analysis to assess the ADME profile and drug-likeness properties of QUR and LIN compared with control DZP, as shown in Figure 7, and the results are summarized in Table 7. This section compiles physicochemical and molecular characteristics.

The drug-likeness feature is critical in determining the similarity of a molecule generating an oral medication in terms of bioavailability [60]. SWISS-ADME offers the Lipinski approach, which indicates that a molecule is classified as drug-like. The Lipinski rule states that a drug-like chemical substance must have a molecular weight (MW) of <500 g/mol, a log *p* value of <5, which indicates that it is hydrophobic, hydrogen-bond donors (HBDs) < 5, hydrogen bond acceptor (HBA) sites < 10, and a polar surface area (PSA) of ≤140 Å [61]. Results indicate that QUR and LIN have drug-likeness properties because they match the Lipinski criteria. Solubility in water is a unique quality in drug development that influences absorption and delivers a good number of active components in a small volume of therapeutic doses [62]. Figure 7 and Table 7 also showed that QUR and LIN are water-soluble and have high GI absorption. Their bioavailability score and synthetic accessibility were also comparable with the standard.

## 3. Discussion

The major inhibitory network in the brain is the GABAergic system, which is crucial for numerous neural processes like neurogenesis, neuronal development, and neuroapoptosis [63]. Abnormality in the GABAergic system can contribute to the pathogenesis of a multitude of mental diseases, including depression, because of the broad spectrum of neurotransmission activity controlled by GABA neurons [64]. To better understand the function of the GABAergic system in the development of depression and anxiety, along with potential treatments, genetic variations in GABA_A_ receptor subtypes are being used more often [65]. Research findings suggest that dysfunction of GABAergic receptors contributes to the onset of depression and that restoration of GABA homeostasis results in the resolution of depressive symptoms [66]. Furthermore, it has been discovered that depressed patients have reduced GABA levels [67]. Stress-related alterations in the brain can lead to depression in humans. For a better analysis of the human brain, researchers have been examining how chronic stress affects neuroplasticity and cognitive performance in rat models [68].

Thiopental sodium (TS), a depressant or sedative, is used in operating rooms as a pre-anesthetic to treat various medical conditions like sleeplessness and seizures [69]. On the other hand, drugs that are sedative and anxiolytic, like DZP, work by binding to the GABA_A_ receptor [70]. It alters physiological sleep pressure after treatment, thus decreasing sleep latency and increasing total sleep time and sleep efficiency [71]. Because brain temperature decreases when behavior moves from active to peaceful waking, the fall in cortical temperature in waking shown after DZP may be caused by a moderate sedative [72]. In this respect, sedatives and CNS depressants reduce sleep latency and stretch sleep, respectively.

Most preclinical in vivo studies use male experimental animals to avoid hormonal interferences in the interventions. However, these male biases may result in variations in the selection of dose, dose frequency, biopharmaceutical, and pharmacokinetic parameters for a whole population. For example, according to the US Food and Drug Administration (FDA), between 1997 and 2000, ten prescription drugs produced severe adverse effects in women. Thus, to obtain precise and reproducible biological outcomes having applicability to both men and women, it is crucial to assess a new drug candidate using both-sexed animals in a preclinical investigation [73]. In the present study, we used both-sexed *Swiss* albino mice.

Findings from this study suggest that both QUR and LIN show dose-dependent sedative effects in TS-induced sleeping mice. However, LIN at all doses exerted better sedative effects than the QUR groups. In contrast, QUR is an edible plant-derived flavonoid that is a well-reported antioxidant. The dietary intake of QUR is estimated at 10–16 mg/day. The daily recommended dosage of its aglycone is 1 g/day [74]. It seems that QUR is well-tolerated in humans. In a recent study, QUR-3-*O*-glucuronide at 150 and 300 mg/kg oral doses exerted significant sedative effects in TS-induced mice [75]. Moreover, a recent review found that QUR and some of its derivatives within the range of 10 to 2000 mg/kg (oral) have significant anti-depressant effects in experimental animals (e.g., mice, rats, and zebrafish) [76]. QUR has a claimed half-life of 11 to 28 h, and human individuals can absorb considerable quantities of the substance via meals or supplements [77]. Results from this investigation showed that QUR at the largest dose (50 mg/kg, p.o.) exhibits the highest latency and the lowest sleep duration in TS-induced mice, which agrees with the antidepressant-like effects observed in previous reports. On the other hand, LIN has an anesthetic effect [78] and can be used to treat depression [79].

Previous in vivo studies reported that the plasma half-life of LIN was just around 45 min, and it was quickly removed from the plasma. After 240 min, LIN in plasma was no longer detectable [80]. In rats, a single oral dosage of 500 mg/kg bw resulted in 97% of LIN being removed from tissues after 72 h [81]. Thus, the sedative-like effect of LIN in this study is in agreement with previous studies. As we aimed to combine the highest response-producing test groups with or without the standard drug DZP, our findings suggest that both QUR and LIN exhibit better effects at the 50 mg/kg oral dose. Therefore, we used their combinations with or without DZP-3. Our in vivo findings suggest that LIN-50 decreases latency and increases sleep duration when co-treated with QUR-50. However, LIN-50, when combined with QUR-50 and DZP-3, significantly increased both parameters in animals. In this case, the observed sleep duration was significantly lower than that of the standard drug DZP-3 as well as the test sample LIN-50 groups. In this respect, certain drugs at low to moderate doses can act as stimulants, while at large doses they may have the opposite effect; for example, kratom [82]. Similarly, sedative drugs at large doses do not result in more rapid or effective sedation but may result in some adverse effects, including anxiety, restlessness, and agitation [83]. Our findings suggest that both LIN-50 and DZP-3 show clear sedative effects in TS-induced animals. Thus, the combined doses of these two sedative agents might produce anxiety, agitation, and restlessness, thereby causing delayed and low sleep in the experimental animals.

**Figure 8:**
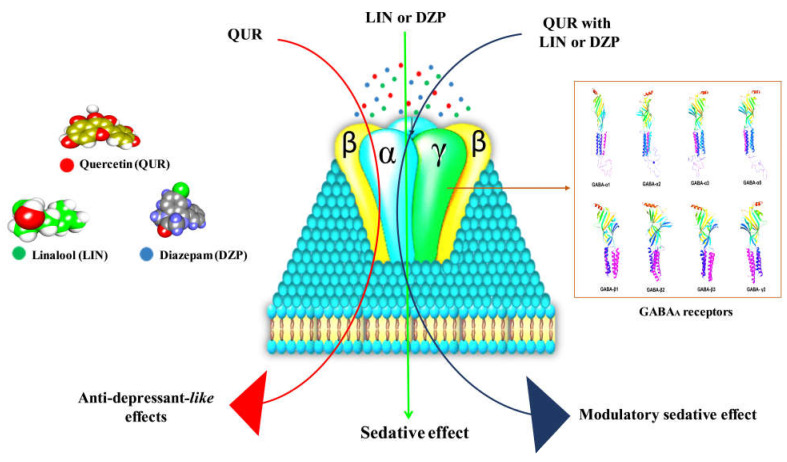
The possible sedation modulatory effects mechanism.

Searching for novel drugs and their development is time-consuming, diverse, and challenging. In this respect, in silico molecular docking has gained in popularity in recent years as a feature of computer-assisted drug design [84]. The fundamental advantage of in silico drug design is that it significantly reduces the cost of drug discovery and development. This approach has the potential to make a significant commitment to all phases of drug manufacturing, from development to completion. The numerous components of fundamental research and application are merged and inspire one another across the vast area of in silico methodologies [85]. The discipline employs cutting-edge approaches such as docking investigations, structure-based design, molecular dynamics, homology modeling, the Ramachandran plot, and increasing biological and chemical information [86]. Our in silico findings suggest that QUR has a high potential for interaction with GABA_A_ receptor subunits α3 (−8.2 kcal/mol), β1 (−8.0 kcal/mol), and γ2 (−7.0 kcal/mol). In contrast, LIN interacted favorably with the GABA_A_ β1 (−5.8 kcal/mol) subunit. Additionally, there are no violations of the Lipinski rule found in the pharmacokinetic analysis of QUR and LIN by SWISS-ADME, suggesting good absorption properties. In this study, QUR and LIN exhibited a sedation-modulatory effect in mouse models. Figure 8 depicts quercetin and linalool putative sedation modulatory mechanisms based on our in vivo and in silico studies.

In conclusion, QUR exerts an anti-depressant-like effect through binding with the GABA_A_ receptor [87,88,89,90,91]. On the other hand, DZP and LIN exert their sedative effects via binding with the GABA_A_ receptor. The results suggest that QUR combined with LIN and/or DZP can antagonize the sedative effects of LIN and DZP [92,93].

## 4. Materials and Methods

### 4.1. In Vivo Study

#### 4.1.1. Chemicals and Reagents

Diazepam (DZP) and thiopental sodium (TS) were kindly provided by ACME Laboratories Ltd. and Square Pharmaceuticals Ltd., respectively. Linalool 97% (LIN) [(±)-3,7-dimethyl-1,6-octadien-3-ol] was purchased from Merck KGaA, Darmstadt, Germany, while quercetin (QUR) and tween-80 [94] were bought from Loba Chemie Pvt. Ltd., Mumbai, Maharashtra 400005, India.

#### 4.1.2. Experimental Animals

Healthy *Swiss* albino mice (*Mus musculus*) (24–28 g) of either sex, purchased from the livestock supply section of Jahangirnagar University in Savar, Dhaka, Bangladesh, were used throughout this investigation. These animals were housed at constant room temperature (27 ± 1 °C) in the pharmacology lab at Bangabandhu Sheikh Mujibur Rahman Science and Technology University (BSMRSTU), Gopalganj. The animals were given free access to conventional food and water and kept under controlled illumination (12 h dark/light cycle) until the start of the experiment. The current study was conducted from 8:00 a.m. to 3:00 p.m., and the animals were observed for an additional 17 h for death following the tests. The experimental design and techniques were performed under standard conditions approved by the BSMRSTU, Department of Pharmacy.

#### 4.1.3. Selection of Test Doses for Quercetin and Linalool

Numerous reports suggest that QUR exerts significant antidepressant effects in experimental animals (e.g., mice, rats, and zebrafish) within 10 to 2000 mg/kg oral doses [95]. On the other hand, the neurobehavioral and genotoxic activities of LIN were studied in mice using 10 to 200 mg/kg intraperitoneal doses [96]. Therefore, we selected the highest dose for each component at 50, 25, and 10 mg/kg oral doses for this current study. When testing hypnotic, sedative, anxiolytic, and other effects on mice and rats, DZP (1 to 3 mg/kg) is frequently used as a standard drug [97,98,99].

#### 4.1.4. Study Design (Thiopental Sodium-Induced Sleeping Test in Mice)

After three days of acclimation, animals were randomly divided into different groups under two squads, each group containing six mice (*n* = 6), as shown in Table 8. The first squad was selected to check the dose-dependent effects of each test compound. In the second squad, the test samples were given at high doses with or without standard drugs to see their possible combined effects. The vehicle was used as a control at 10 mL/kg (p.o.), while diazepam (3 mg/kg, po) was used as a reference drug. Then, each animal was given thiopental sodium (TS) (10 mg/kg, ip) to induce sleep after 0.5 h of the treatments before being placed in an observation chamber (e.g., a plastic cage). Once the righting reflex was lost after TS administration, the latent period was recorded. ‘First squad’ means non-combined groups of tests or controls, while ‘Second squad’ means combined treated groups (highest response producing test groups with or without DZP-3 group). Combined treatments were administered solely and one by another maintaining a 2 min gap between each treatment. The time that passed while sleeping between the loss and recovery of the reflex (sleep duration) was also recorded. The percentages of sleep incidence and modulation (increase or decrease) of latency or sleep duration were calculated using the following equation:

$$\%\mathrm{Incidence}\;\mathrm{of}\;\mathrm{sleep}=\frac{\mathrm{Number}\;\mathrm{of}\;\mathrm{slept}\;\mathrm{mice}}{\mathrm{Total}\;\mathrm{mice}\;\mathrm{in}\;\mathrm{the}\;\mathrm{group}}\times 100$$

$$\%\mathrm{Decrease}\;\mathrm{in}\;\mathrm{latency}=\frac{\mathrm{Latency}\;\mathrm{of}\;\mathrm{control}\;\mathrm{group}-\mathrm{Latency}\;\mathrm{of}\;\mathrm{test}\;\mathrm{group}}{\mathrm{Latency}\;\mathrm{of}\;\mathrm{control}\;\mathrm{group}}\times 100$$

$$\%\mathrm{Increase}\;\mathrm{in}\;\mathrm{sleep}\;\mathrm{duration}=\frac{\mathrm{Sleeping}\;\mathrm{time}\;\mathrm{of}\;\mathrm{test}\;\mathrm{group}-\mathrm{Sleeping}\;\mathrm{time}\;\mathrm{of}\;\mathrm{control}\;\mathrm{group}}{\mathrm{Sleeping}\;\mathrm{time}\;\mathrm{of}\;\mathrm{test}\;\mathrm{group}}\times 100$$

#### 4.1.5. Statistical Analysis

All determinations were conducted in triplicate, and the data were subjected to a one-way analysis of variance (ANOVA). Results are expressed as the mean ± standard error of the mean deviation (S.E.M.). Statistical analysis was performed with the aid of Student–Newman–Keuls post hoc test using GraphPad Prism (version 9.5) [100] (GraphPad Software, San Diego, California, USA, http://www.graphpad.com (accessed on 18 November 2022), and the experimental groups were compared to the vehicle (control) group; differences were considered significant at *p* ≤ 0.05 at 95% confidence intervals.

### 4.2. Molecular Docking (In Silico) Study

#### 4.2.1. GABA Homology Model

##### Retrieval of Sequence

The UniProt protein database was used to retrieve the protein sequence of human GABA_A_ subunits, which was then stored in Notepad in the FASTA file format with the accession ID. The amino acid sequences of proteins are contained in the publicly available protein database known as UniProt [101].

##### Model Building and Evaluation

The SWISS-MODEL website was used to do comparative homology modeling on the GABA protein sequences. A completely automated service for protein-structure-homology modeling called SWISS-MODEL makes protein models available to all biotechnologists [102]. To choose the template, BLAST analysis was carried out using the NCBI BLAST tools [103]. The Phi/Psi Ramachandran plot assessed the stereochemical characteristics of the projected models [104]. Furthermore, the quality and validity of the models were checked using the PROCHECK technique using the PDBsum server [105].

#### 4.2.2. Protein Preparation

PyMOL version 1.7.4.5 Edu was used to eliminate unnecessary amino acid residues and water molecules to refine the macromolecules [106]. After that, the SWISS-PDB Viewer software program (version 4.1.0) with the GROMOS 96 43B1 parameters set was used to minimize the energy consumption of the protein structures before docking [107].

#### 4.2.3. Ligand Preparation

The chemical formulas for quercetin (QUR) (PubChem ID: 5280343), linalool (LIN) (PubChem ID: 6549), and the standard drug diazepam (DZP) (PubChem ID: 3016) (Figure 1) were taken from the PubChem chemical database in the ‘sdf’ file format. The Allinger’s force field (MM2) method was used for energy minimization of the ligands by Chem3D Pro21.0 software [108].

#### 4.2.4. Docking Protocol and Non-Bond Interactions

A computerized drug design method in drug discovery is essential when computing docking study simulations. Through the examination and placement of molecules at specific binding sites, the PyRx (version 0.8) virtual screening tool was used to perform the molecular docking study [109]. Docking results indicate the degree of binding to a target molecule’s catalytic site. Kcal/mol was used to measure the ligand’s binding affinity as a unit for a negative score [110]. BIOVIA Discovery Studio version 2021 was employed to investigate the bonding interactions of the ligand–protein complexes [111].

#### 4.2.5. Pharmacokinetics and Drug-Likeness Properties

The pharmacokinetic study looks at how the body responds to drugs that are gradually administered. The in silico ADME strategy is an excellent method that initially evaluates the pharmacokinetic features of a chemical before converting it into a useful medicine [112]. In the discovery and development of drugs, the “drug-likeness” property is used to assess how closely a molecule resembles a medicine. The SWISS-ADME system [113] was used to investigate the drug-like properties of QUR and LIN and the pharmacokinetic activities of ligands.

## 5. Conclusions

Several problems known as sleep disorders hinder a person from getting enough sleep, and depression is a highly prevalent and challenging mental illness that impacts emotional health. To address these conditions, several conventional drugs with neuroprotective characteristics are available. Findings from this study suggest that DZP and LIN exert dose-dependent sedative effects on TS-induced sleeping mice. QUR showed dose-dependent antidepressant-like effects in TS-induced mice. However, QUR-50, when combined with LIN-50 and/or DZP-3, significantly increased the latency while decreasing the sleep duration in animals compared to individual LIN and DZP groups, suggesting better effects in their combination. Furthermore, our in silico investigation indicates that QUR and LIN interact better with GABA_A_ α3, β1, and γ2 subunits than other GABA_A_ receptor subunits which are similar to the standard DZP. These compounds also provide a good ADME score. We presume that QUR might have antagonistic sedative effects with LIN and DZP through the interaction with selective GABA receptors, especially with its α3, β1, and γ2 subunits.

## Figures and Tables

**Table 1:** Latency, duration, and incidence of sleep were observed in the test and control groups.

First Squad			
Treatment Group	Latency (min)	Sleeping Time (min)	Sleep Incidence (%)
Gr-I	5.40 ± 0.84	41.20 ± 5.02	100
Gr-II	3.20 ± 0.65 *	99.60 ± 5.73 *	100
Gr-III	29.40 ± 2.08	12.40 ± 2.21	100
Gr-IV	42.60 ± 2.01	15.60 ± 1.23	100
Gr-V	53.40 ± 2.56	24.00 ± 5.24	100
Gr-VI	5.80 ± 0.73	44.60 ± 2.01 *	100
Gr-VII	6.80 ± 0.97	46.40 ± 2.01 *	100
Gr-VIII	8.40 ± 0.76	53.20 ± 4.04 *	100

Values are the Mean ± SEM (standard error of the mean) (*n* = 6); One-way ANOVA followed by *t*-Student–Newman–Keuls’s as a posthoc test; * *p* < 0.05 compared to the vehicle group; Gr-I: vehicle; Gr-II: diazepam (DZP) 3 mg/kg; Gr-III: quercetin (QUR) 10 mg/kg; Gr-IV: QUR-25 mg/kg; Gr-V: QUR-50 mg/kg; Gr-VI: Linalool (LIN) 10 mg/kg; Gr-VII: LIN-25 mg/kg; Gr-VIII: LIN-50 mg/kg.

**Table 2:** Latency, duration, and incidence of sleep were observed in the test and/or control groups.

Second Squad			
Treatment Group	Latency (min)	Sleeping Time (min)	Sleep Incidence (%)
Gr-CI *	5.40 ± 0.84	41.20 ± 5.02	100
Gr-CII ^a^	53.40 ± 2.56	24.00 ± 5.24	100
Gr-CIII ^b^	8.40 ± 0.76 ^a^	53.20 ± 4.04 *^a^	100
Gr-CIV	3.20 ± 0.65 *^ab^	99.60 ± 5.73 *^ab^	100
Gr-CV	16.80 ± 2.43 ^a^	34.00 ± 5.46 ^a^	100
Gr-CVI	43.00 ± 4.15 ^a^	46.20 ± 1.30 *^a^	100

Values are Mean ± SEM (standard error of the mean) (*n* = 6); One-way ANOVA followed by Student–Newman–Keuls as a post hoc test; * *p* < 0.05 compared to the vehicle group; ^a^ *p* < 0.05 compared to Gr-CII; ^b^ *p* < 0.05 compared to Gr-III; Gr-CI: vehicle; Gr-CII: quercetin (QUR) 50 mg/kg; Gr-CIII: linalool (LIN) 50 mg/kg; Gr-CIV: diazepam (DZP) 3 mg/kg; Gr-CV: (QUR-50 + LIN-50) mg/kg; Gr-CVI: (QUR-50 + DZP-3 + LIN-50) mg/kg.

**Table 3:** The percentage modulation of latency and sleep duration in the standard groups compared to the vehicle (control) is shown in Table 2.

Treatment Group	Latency Decrease (%)	Sleeping Time Increase (%)
Gr-CII	-	-
Gr-CIII	-	22.56
Gr-CIV	92.23	58.63
Gr-CV	-	-
Gr-CVI	-	10.82

Values are percentage increase/decrease compared to the control (Gr-CI: vehicle) group; Gr-CII: Quercetin (QUR) 50 mg/kg; Gr-CIII: linalool (LIN) 50 mg/kg; Gr-CIV: diazepam (DZP) 3 mg/kg; Gr-CV: (QUR-50 + LIN-50) mg/kg; Gr-CVI: (QUR-50 + DZP-3 + LIN-50) mg/kg.

**Table 4:** The molecular docking study of QUR with GABA_A_ (α1, α2, α3, α5, β1, β2, β3, and γ2) receptors subunits.

Protein (Receptor)	Binding Affinity (Kcal/mol)	Number of Hydrogen Bond	Number of Hydrophobic Bond	Number of Others Bond
GABA_A_ α1	−7.1	2	4	-
GABA_A_ α2	−7.9	5	5	1
GABA_A_ α3	−8.2	2	5	-
GABA_A_ α5	−7.5	2	2	1
GABA_A_ β1	−8.0	4	4	-
GABA_A_ β2	−7.8	4	2	-
GABA_A_ β3	−7.0	5	2	-
GABA_A_ γ2	−7.0	4	3	-

**Table 5:** The molecular docking study of linalool (LIN) with GABA_A_ (α1, α2, α3, α5, β1, β2, β3, and γ2) receptor subunits.

Protein (Receptor)	Binding Affinity (Kcal/mol)	Number of Hydrogen Bond	Number of Hydrophobic Bond	Number of Others Bond
GABA_A_ α1	−4.8	1	11	-
GABA_A_ α2	−4.5	-	6	-
GABA_A_ α3	−5.2	1	9	-
GABA_A_ α5	−4.8	1	7	-
GABA_A_ β1	−5.8	-	12	-
GABA_A_ β2	−4.8	1	6	-
GABA_A_ β3	−4.8	1	6	-
GABA_A_ γ2	−4.3	-	7	-

**Table 6:** The molecular docking study of the standard drug, diazepam (DZP) with GABA_A_ (α1, α2, α3, α5, β1, β2, β3, and γ2) receptor subunits.

Protein (Receptor)	Binding Affinity (Kcal/mol)	Number of Hydrogen Bond	Number of Hydrophobic Bond	Number of Others Bond
GABA_A_ α1	−6.4	1	7	1
GABA_A_ α2	−6.7	1	7	1
GABA_A_ α3	−6.8	-	9	-
GABA_A_ α5	−6.5	3	4	3
GABA_A_ β1	−7.8	1	6	-
GABA_A_ β2	−7.0	2	2	2
GABA_A_ β3	−6.3	2	2	-
GABA_A_ γ2	−7.7	5	2	-

**Table 7:** The pharmacokinetic profile and drug-likeness properties of quercetin (QUR), linalool (LIN), and standard drug, diazepam (DZP).

Properties	Factors	Quercetin	Linalool	Diazepam
Physico-chemical properties	Formula	C_15_H_10_O_7_	C_10_H_18_O	C_16_H_13_ClN_2_O
	MW (g mol^−1^)	302.24	154.25	284.74
	Heavy atoms	22	11	20
	Arom. heavy atoms	16	0	12
	H-Bond acceptors (HBAs)	7	1	2
	H-Bond donors (HBDs)	5	1	0
	Molar refractivity	78.03	50.44	87.95
	TPSA (Å^2^)	131.36	20.23	32.67
Lipophilicity	log Po/w (XLOGP3)	1.54	2.97	2.99
Water solubility	log S (ESOL)	Soluble	Soluble	Soluble
Pharmacokinetics	GI absorption	High	High	High
Drug likeness	Lipinski	Yes	Yes	Yes
	Bioavailability score	0.55	0.55	0.55
Medicinal chemistry	Synthetic accessibility	3.23	2.74	3.00

**Table 8:** Group division, name of treatment, and dosage.

First Squad		
Treatments	Composition	Dose
Gr-I	Vehicle (0.5% tween 80 dissolved in normal saline)	10 mL/kg
Gr-II	Diazepam (DZP)	3 mg/kg
Gr-III	Quercetin (QUR)	10 mg/kg
Gr-IV	QUR	25 mg/kg
Gr-V	QUR	50 mg/kg
Gr-VI	Linalool (LIN)	10 mg/kg
Gr-VI	LIN	25 mg/kg
Gr-VI	LIN	50 mg/kg
Second squad		
Treatments	Composition	Dose
Gr-CI	Vehicle	10 mL/kg
Gr-CII	QUR	50 mg/kg
Gr-CIII	LIN	50 mg/kg
Gr-CIV	DZP	3 mg/kg
Gr-CV	QUR-50 + LIN-50	50 mg/kg + 50 mg/kg
Gr-CVI	QUR-50 + DZP-3 + LIN-50	50 mg/kg + 3 mg/kg + 50 mg/kg

All treatments are given at 10 mL/kg via oral gavage (p.o.) (*n* = 6).

## Data Availability

Not applicable.
